# Molecular Analysis of Vancomycin-Resistant Enterococci Isolated from Regional Hospitals in Trinidad and Tobago

**DOI:** 10.1155/2016/8762691

**Published:** 2016-05-19

**Authors:** Patrick E. Akpaka, Shivnarine Kissoon, Padman Jayaratne

**Affiliations:** ^1^Department of Paraclinical Sciences, Faculty of Medical Sciences, The University of the West Indies, St. Augustine Campus, St. Augustine, Trinidad and Tobago; ^2^Department of Pathology and Molecular Medicine, McMaster University, 1280 Main Street W., Hamilton, ON, Canada L8S 4L8

## Abstract

Geographic spread of vancomycin-resistant enterococci (VRE) clones in cities, countries, or even continents has been identified by molecular techniques. This study aimed at characterizing virulent genes and determining genetic relatedness of 45 VRE isolates from Trinidad and Tobago using molecular tools, including polymerase chain reaction, pulsed-field gel electrophoresis (PFGE), and Random Amplification Polymorphic DNA (RAPD). The majority (84%) of the isolates were* Enterococcus faecium* possessing* van*A gene while the rest (16%) were* Enterococcus faecalis* possessing* van*B. The* esp* gene was found in all 45 VRE isolates while* hyl* genes were found only in* E. faecium* species. The* E. faecium* species expressed five distinct PFGE patterns. The predominant clones with similar or common patterns belonged to clones one and three, and each had 11 (29%) of the VRE isolates. Plasmid content was identified in representative isolates from each clonal group. By contrast, the* E. faecalis* species had one PFGE pattern suggesting the presence of an occult and limited clonal spread. The emergence of VRE in the country seems to be related to intra/interhospital dissemination of an epidemic clone carrying the* van*A element. Therefore, infection control measures will be warranted to prevent any potential outbreak and spread of VRE in the country.

## 1. Introduction

Vancomycin-resistant enterococci (VRE) were first described in Great Britain in 1988 and shortly afterwards were reported in other European countries and the USA [1, 2]. In Latin America, VRE have been reported in Brazil, Colombia, and Argentina [3]. Several reports of outbreaks and spread in hospitals, communities, nursing homes, and long term care institutions have been documented [3]. Epidemiologic links of VRE clones occurring in different hospitals, countries, and regions have been demonstrated in several places [3]. Understanding disease mechanisms, organism's virulence, and host predisposition must be considered. Enterococcal surface protein encoded by the* esp* gene is a virulence factor found in both* Enterococcus faecalis* and* Enterococcus faecium*. In addition, the presence of the variant* esp* gene in* E. faecium* was reported to be associated with in-hospital spread whereas the hyaluronidase (*hyl*) gene was also regarded as a potential virulence gene associated with invasive disease [4, 5]. Although the prevalence rate of VRE in Trinidad and Tobago is low, 3.9% [6], there are no molecular analysis or epidemiologic reports of VRE isolates available in the country.

Understanding the molecular epidemiology of VRE is crucial for assessing and implementing infection control measures in any healthcare institution or country [7]. The aim of this study was to detect the phenotypes and genotypes of vancomycin resistance, their plasmid contents, virulence factors, analysis of the* esp* repeat profile, and molecular relatedness among enterococci isolated from hospitals in Trinidad and Tobago. This information may provide the background level of VRE in Trinidad and Tobago and be of help in controlling nosocomial spread if a VRE outbreak occurs.

## 2. Materials and Methods

The 45 VRE bacterial isolates used for this analysis were those identified among 1,141 enterococcal isolates from previously reported study [6]. These isolates were from five regional hospitals (tagged as A–E) in the country and were identified phenotypically by standard microbiologic laboratory procedures [8]. No duplicate isolates from a single patient were included and there was no history of VRE outbreak during the study period.

The antimicrobial susceptibility tests were performed by the standard disk diffusion method and minimum inhibitory concentration (MIC) determined using the Microscan WalkAway 96 SI (Siemens, USA).* Staphylococcus aureus* ATCC 25923 and* E. faecalis* ATCC 29212 strains were used as controls. The antibiotics ampicillin, ciprofloxacin, levofloxacin, linezolid, nitrofurantoin, penicillin, quinupristin/dalfopristin, rifampicin, tetracycline, and vancomycin included in the Gram-positive panel 20 of the Microscan were tested. The MIC values were interpreted according to approved CLSI breakpoints [9] as previously reported [6]. The MIC values of the enterococcal isolates were as follows for the antibiotics: AMP ≤ 8 *μ*g/mL; CIP ≤ 1 *μ*g/mL; LEV ≤ 2 *μ*g/mL; LZD ≤ 2 *μ*g/mL; PEN ≤ 8 *μ*g/mL; Q-D ≤ 1; RIF ≤ 1 *μ*g/mL; TET ≤ 4 *μ*g/mL; VAN ≤ 8 *μ*g/mL.

### 2.1. Multiplex Polymerase Chain Reaction (PCR)

Determination of glycopeptide resistance genotypes and confirmation of species identification were performed by multiplex polymerase chain reaction (PCR), as previously described by Jayaratne and Rutherford [10]. Briefly, prepared bacteria cells in normal saline mixed in lysis buffer were subjected to PCR amplification in 50 *μ*L reaction mixtures containing deoxynucleoside triphosphate, two primers (*van*A: forward, 175-GGGAAAACGACAATTGC-191; reverse, 907-GTACAATGCGCCGTTA-891;* van*B: forward, 173-ATGGGAAGCCGATAGTC-189; reverse, 807-GATTTCGTTCCTCGACC-791), Taq polymerase, MgCl_2_, buffer, and H_2_O.

The samples were subjected to 30 PCR cycles, each consisting of one minute of denaturation at 94°C, one minute of annealing at 58°C, and one minute of elongation at 72°C. PCR products were analyzed by electrophoresis on 1% agarose gels and were stained with ethidium bromide.

**Figure 1:**
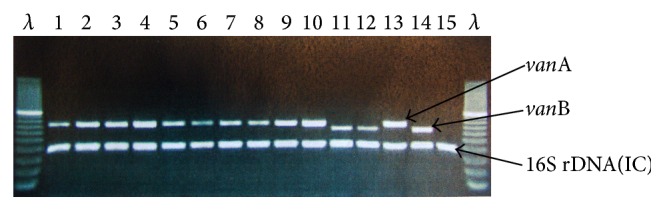
Agarose gel electrophoresis of PCR amplified products of vancomycin-resistant enterococci (VRE) isolates obtained from Trinidad and Tobago in 2008 to 2012. Lane *λ* is the markers. Lanes one–ten represent* van*A positive* E. faecium* and lanes 11 and 12 represent* van*B positive* E. faecalis* from Trinidad and Tobago. Lanes 13–15 represent control strains of* van*A (*E. faecium* ATCC 700221), with* van*B (*E. faecalis* ATCC 51299) and* Enterococcus* vancomycin-sensitive (*E. faecalis* ATCC 29212), and 16S rDNA is the Internal Amplification Control (IC), respectively.

A* van*A strain (*E. faecium* ATCC 700221), a* van*B strain (*E. faecalis* ATCC 51299), and a vancomycin susceptible* E*.* faecalis* (ATCC 29212), 16S rDNA Internal Amplification Control, were run with each set of reactions as quality positive and negative controls (Figure 1).

#### 2.1.1. Pulsed-Field Gel Electrophoresis (PFGE)

Genomic DNA was prepared in agarose plugs as described by Murray et al. and Turabelidze et al. with some modifications [11, 12]. After cell lysis by mutanolysin in lysoenzyme and incubation with proteinase, the DNA was digested with* Sma*1. The PFGE was performed using a contour-clamped homogenous electric field apparatus (CHEF DRIII, Bio-Rad Laboratories, Hercules, CA, USA). Gel images were captured on the Gel Doc imaging system using Quality One Software version 4.4.1 (Bio-Rad Laboratories, Hercules, CA, USA). The resulting banding patterns were analyzed by visual inspection according to previously established criteria [13, 14]. Gel analysis was performed using Bionumerics version 3.5 (Applied Maths, Austin, TX, USA) and cluster analysis was achieved using Dice coefficient and UPGMA.

#### 2.1.2. Random Amplified Polymorphic DNA (RAPD)/PCR Amplification

PCR assays were routinely performed in a 25 *μ*L reaction mixture containing 20–30 g of genomic DNA, 2.5 *μ*L 10x buffer, one-unit Taq DNA polymerase, two *μ*mol primer, one mmol each of dCTP, dGTP, dATP, and dTT, and 2.5 mmol MgCl_2_. AP4 (5′ TCA CGC TGC A-3′) random primer was used for RAPD. PCR reactions were performed on Perkin Elmer 9600 under the following conditions: 30 cycles of 94°C for one minute, 36°C for one minute, and 72°C for two minutes, with a final extension of 72°C for five minutes. PCR products were run on 1.5% agarose gels and stained with ethidium bromide. DNA ladder (Promega, USA) was used as DNA size markers. AP4 (5′ TCA CGC TGC A-3′) primers were chosen for RAPD analysis because on PCR they yielded clear patterns [15].

#### 2.1.3. Repetitive-Sequence-Based-PCR (Rep-PCR)

Repetitive-sequence-based-PCR (Rep-PCR) methods are rapid typing procedures that amplify the regions between the noncoding repetitive sequences in bacterial genomes [16]. The ERICIR (5′ ATG TAA GCT CCT GGG GAT TCA C-3′) was used for Rep-PCR. The genetic relatedness of VRE isolates was determined by Rep-PCR typing as previously described in Healy et al. [17]. DNA was extracted using a one *μ*L loop of plated culture or one mL of broth culture and the Ultraclean Microbial DNA Isolation Kit (Mo Bio Laboratories, Solana Beach, Calif.) following the manufacturer's instructions. The extracted DNA was amplified using the DiversiLab* Enterococcus* fingerprinting (Spectral Genomics, Inc., Houston, TX) according to the manufacturer's instructions. Genomic DNA, the Rep-PCR primer (*Enterococcus*) Ampli*Taq*, and PCR buffer (Applied Biosystems) were all mixed together and subjected to thermal cycling. Amplicons were separated by 1.5% agarose gel electrophoresis (gels, 25 by 25 cm^2^) containing ethidium bromide (3 *μ*g/mL in gel and in 1x tris-acetate-EDTA running buffer) for six hours at 120 V in a recirculating electrophoresis unit. DNA ladder (Promega, USA) was used as DNA size markers. Gel images were captured on the Gel Doc imaging system using Quality One Software version 4.4.1 (Bio-Rad Laboratories, Hercules, CA, USA).

### 2.2. Detection of* esp* and* hyl* Genes by PCR

The presence of* esp* and* hyl* genes was determined for all 45 VRE isolates by PCR as described by Vankerckhoven et al. [18, 19]. Bacteria cultures grown on Columbia agar (Becton Dickinson, MD) supplemented with 5% sheep blood were incubated at 37°C. Bacterial DNA suspension, 0.1 *μ*m of primer* hyl* and 0.2 *μ*m of* esp* primer including HotStar Taq Master Mixture (Qiagen, Hilden, Germany), Taq DNA polymerase, and deoxynucleoside triphosphates were all subjected to 30 PCR cycles. The PCR products were analyzed by electrophoresis on 1.5% pronarose gel for one hour at 150 V and were stained with ethidium bromide.* E. faecium* strain C68 (*hyl*_Efm_ and *esp*_Efm_) was used as the positive control. A 100 bp DNA ladder (Bio-Rad) was used as a molecular size marker.

#### 2.2.1. Determination of Variation in the* esp* A and C Repeats

For determining repeat number variations of* esp* A and C repeats, two different primer combinations were used: esp_fs_ 7F-esp_fm_ 5R and esp_fs_ 5F-esp_fm_ 3R, respectively [17]. Briefly, chromosomal DNA was purified as described elsewhere [11]. PCR conditions for all amplification reactions were performed in 25 *μ*L volumes with HotStar Taq Polymerase and HotStar Master Mix buffers (Qiagen Inc., Valencia, CA). Subsequently, the amplicons were subjected to agarose gel electrophoresis (1%) in order to determine their sizes. From the sizes of the amplicons the numbers of repeats were deduced. Amplicon size differences corresponded to multiples of either 252 bp (A repeats) or 246 bp (C repeats).

#### 2.2.2. Determination of Plasmid Content

A subset of isolates representing at least one isolate indicative of each major PFGE pattern was assessed for the presence of the plasmids by the SI nuclease method as described by Barton et al. [20].

## 3. Results

All 45 VRE isolates used for this analysis and their hospital and facility distribution were from work previously reported [6]. More than half (54%, 24/45) of the VRE isolates were recovered from urogenital tract system infections, and 42.2% (19/45) were from skin and soft tissue infections (Table 1). One isolate each was recovered from blood and gastrointestinal tract, respectively. The GIT isolate was from peritoneal fluid of a patient who had peritonitis. All isolates (100%) were from hospitalized patients and thus represented healthcare-associated isolates and infections. Most (84%, 38/45) of the isolates were* E. faecium* and the rest (16%, 7/45) are* E. faecalis*. All enterococcal isolates had an MIC value for vancomycin ≥ 32 *μ*g/mL; they all were 100% susceptible to linezolid. Although only 18% of the* E. faecium* were resistant to quinupristin-dalfopristin but 100% resistant to ciprofloxacin, erythromycin, and levofloxacin, all* E. faecalis* were 100% resistant to ciprofloxacin, erythromycin, levofloxacin, and quinupristin-dalfopristin.

All the* E*.* faecium* isolates possessed the* van*A genes while all* E. faecalis* possessed the* van*B genes. Overall, the* esp* gene was detected in all (100%) VRE isolates. None of the isolates had* hyl* genes. Analysis of the* esp* repeat profiles produced similar results. The numbers of A and C repeats of the* esp* gene seen in the* E. faecium* isolates varied from three to seven and from three to eight, respectively. Based on the* esp* A and C repeat profile, these* E. faecium* isolates belonged to five different groups. The most prevalent* esp* profile was A6-C5 (28.9%, 11/38 isolates), followed by A5-C6 (23.9%, 9/38 isolates), A6-C3 (15.8%, 6/38 isolates), A4-C5 (13.2%, 5/38 isolates), and A5-C7 (10.5%, 4/38 isolates).

### 3.1. PFGE Typing

**Figure 2:**
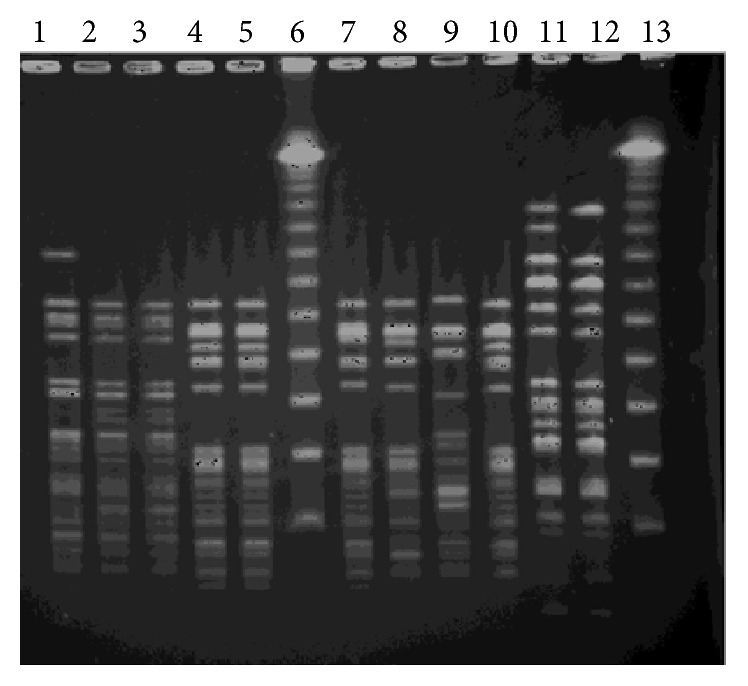
* Sma*1 PFGE profiles of vancomycin-resistant* E. faecium* from major regional hospitals in Trinidad and Tobago, 2008–2012. Lanes one to five and seven to 12 are representative of vancomycin-resistant* E. faecium* isolates from Trinidad and Tobago regional hospitals. Lambda (*λ*) DNA PFGE molecular size marker is indicated in marker lanes 6 and 13. Lane one = PFGE-1 or clone one; lanes two and three = PFGE-2 or clone two; lanes four, five, seven, eight, and ten = PFGE-3 or clone three; lane nine = PFGE-4 or clone four; lanes 11 and 12 = PFGE-5 or clone five.

**Figure 3:**
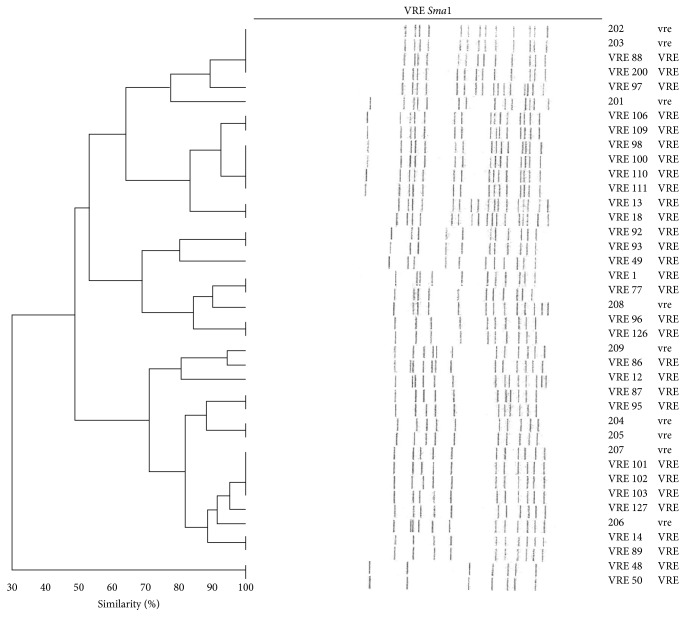
Dendrogram of PFGE vancomycin-resistant* E. faecium*. Molecular typing of vancomycin-resistant* E. faecium* from regional hospitals in Trinidad and Tobago. The phylogenetic tree was constructed by use of the Dice coefficient and UPGMA clustering; the band tolerance was set at 1.5%, and the threshold cut-off value was set at 85%.

The analysis of molecular typing demonstrated five PFGE patterns (Figure 2) among the 38 vancomycin-resistant* E*.* faecium* isolates. The predominant clones were one and three (PFGE-1 and PFGE-3), and each clone occurred in 11 (29%) isolates, respectively. Clone one was present in two hospitals: “D” and “C” hospitals located in the southern and northern geographic areas of Trinidad. Clone three was present in four of five hospitals, “A,” “C,” “D,” and “E,” in the country. Clones two and five (PFGE-2 and PFGE-5) were represented by six (16%) and eight (21%) isolates, respectively, from the two hospitals “C” and “D.” Clone four (PFGE-4) had two isolates, one from “C” and the other from “A” hospitals and both from the urogenital tract (UGT). All the seven vancomycin-resistant* E. faecalis* had an identical PFGE pattern indicating they belong to the same clone (PFGE result not shown). The cluster analysis was achieved by the Bionumerics software (Applied Maths, Austin, TX, USA). Percentages of similarity were determined using the Dice correlation coefficient and a dendrogram (Figure 3) was produced via the unweighted pair group method with arithmetic mean clustering (UPGMA).

RAPD produced concordant patterns to PFGE. Five different RAPD and Rep-PCR types were obtained for the 38* E. faecium* strains. Seven* E. faecalis* isolates showed one Rep-PCR type. The five Rep-PCR types were as follows: type one (29%, 11 isolates), type two (15.8%, 6 isolates), type three (29%, 11 isolates), type four (5.2%, 2 isolates), and type five (21%, 8 isolates). The vancomycin-resistant* E. faecalis* had one RAPD and Rep-PCR type and all had similar banding patterns. These results were concordant with PFGE types which were visually inspected according to Tenover et al. criteria [13].

### 3.2. Plasmid Results

Seven VRE isolates representing the various clones and from different hospitals were randomly chosen for plasmid determination. All the isolates examined had at least one plasmid ranging in size from 48.5 kbp to 200 kbp. One isolate from hospital “C” had multiple plasmids.

## 4. Discussions

In the current study, the genetic characteristics of all vancomycin-resistant enterococci were investigated by PFGE, RAPD, Rep-PCR, and* esp* repeat profiles. Each method had a different ability to analyze the genotypes of VRE isolates.

*E. faecium* remained as in many countries the most prevalent species among VRE (86% of the isolates) which is similar to reports from North America, Australia, and Italy, where prevalence ranged from 79.5% to 99% [21–23]. The majority of vancomycin-resistant* E. faecium* from this current study were multiply resistant to antibiotics such as ciprofloxacin, erythromycin, levofloxacin, rifampicin, and tetracycline, similar to results from other places [22, 23]. In contrast, vancomycin-resistant* E*.* faecalis* showed high (100%) resistance to erythromycin, vancomycin, and quinupristin/dalfopristin. Similar results were observed by Corso et al. [3]. This high resistance observed was probably due to prior exposure and high consumption and usage of antibiotics as previously reported [6].

All vancomycin-resistant* E. faecium* isolates in this study were of the* van*A genotype which showed a high vancomycin MIC of ≥32 *μ*g/mL. A similar high vancomycin MIC has been reported in the United States and Europe [24]. The predominance also of* van*A* E. faecium* in our study is similar to findings in Northern Asia, Europe, and the United States [24, 25]. The predominance of* van*B* E. faecalis* in our study is also similar to findings from Australia and Taiwan where* van*B gene is more common [24].

This present study found the* esp* gene to be present in all the 45 VRE isolates. Although reports of* esp* prevalence vary according to region and population, our results are consistent with those of Shankar et al., who found the prevalence of* esp* to be 77% in a sample of* E*.* faecium* (predominantly* van*A) from eight European countries [5, 26]. Also in the USA, UK, and Spain there have been various prevalence reports ranging from 61% to 70% [26]. The* esp* gene is part of a putative pathogenicity island considered to be a marker for epidemicity and could putatively contribute to the spread of vancomycin-resistant* E*.* faecium* isolates in hospitals [27–29]. The presence of* esp* genes was not associated with the invasiveness or outbreak potential of VRE. Shankar et al. reported that* esp* genes can be deleted from the pathogenicity island of vancomycin-resistant* E*.* faecalis* at a high frequency [30]. Oancea et al. also demonstrated that the* esp* gene is transferable by conjugation among enterococcal isolates [31].

The* esp* repeat profiles used to analyze the VRE isolates revealed that the* E. faecium* strains in the different groups had identical* esp* repeat profiles which were relatively stable and this was similar to other studies by Leavis et al. [19]. The* esp* repeat profiles could be utilized to investigate the outbreaks of resistant clones in combination with other genotyping methods [19].

In this study, the PFGE and the* esp* gene repeat profiles showed multiple genotypes of* E. faecium* isolates which were consistent with the result of RAPD and Rep-PCR. The presence of a dominant vancomycin-resistant* E*.* faecium* clone (clones 1 and 3) in several major hospitals shows that their spread has occurred not only within individual hospitals but also between hospitals of various geographic locations in the country. Other studies have documented the spread of vancomycin-resistant* E*.* faecium* and* E*.* faecalis* clones among hospitals [32, 33]. The spread of clones in different institutions in the country may suggest that some strains contain bacterial factors that enhance their spread within hospitals. Some other researchers [5, 28] have identified the* esp* gene encoding a surface protein associated with virulence for* E. faecalis* and* E. faecium* residing on a pathogenicity island. Studies by Harrington et al. support the hypothesis that a combination of vancomycin resistance and the* esp* gene could lead to dissemination of particular clones [34]. The finding of no* hyl* gene in clinical VRE isolates in this current study suggests a low prevalence or nonexistence of this gene in Trinidad and Tobago. This will definitely be a sharp contrast to the prevalence of* hyl* gene that varies from 3% to 71% among European VRE [4, 18].

In our study, molecular typing results indicate the dominant dissemination of vancomycin-resistant* E. faecium* clones one and three in different wards of the same hospital, in different hospitals, and in different cities. The reason for this may be due to the absence of an alert system for patients infected or colonized with vancomycin-resistant enterococci in the hospitals. There are equally no consistent effective screening mechanisms or policies in place for VRE infections or colonization, and all these could contribute to this dissemination. The isolates in this study were polyclonal with two major clones, suggesting a highly diverse population of hospital acquired* E*.* faecium* strains. This picture can possibly be explained by exchange of a mobile resistance determinant between various enterococci as reported in other places [35–37].

Five different RAPD types were obtained for the 38 vancomycin-resistant* E. faecium* isolates. This demonstrates that* E*.* faecium* strains could be easily differentiated by RAPD fingerprinting, thus supporting the validity of this fast and accurate technique in studying diversity of* E*.* faecium* population [38]. This result is in agreement with findings by Quednau et al. (1999) who reported genetic variability within* E. faecium* [39]. During an outbreak, the identity of isolates should ideally be confirmed by two different methods. Although RAPD requires testing of the reproducibility of the patterns, this technique is easier to perform and less time-consuming than other phenotyping or genotyping techniques proposed for enterococci [38, 39]. Therefore, the RAPD method with AP4 plus ERICIR primers is a powerful tool for microbiologists to investigate VRE isolates in cases of nosocomial infection [15].

The Rep-PCR has been reported to have good typeability and reproducibility [17] and has been used to investigate several nosocomial outbreaks [40]. Comparable findings have been reported for Rep-PCR and PFGE for* Acinetobacter baumannii*,* Streptococcus pneumoniae*, and methicillin-resistant* Staphylococcus aureus* (MRSA) [41]. Rep-PCR may be more suitable as a rapid screening method to exclude the possibility of clonal spread and to facilitate prompt intervention for outbreaks, whereas PFGE could be reserved for confirmation. Using both methods simultaneously could be costly. In areas where there is low VRE prevalence and low clonal spread as in this present study, Rep-PCR may be used as an ideal quick screening tool [42]. The isolates analyzed in this study by Rep-PCR and PFGE showed good reproducibility; the Rep-PCR was highly correlated with PFGE typing to evaluate the clonal spread of VRE in this study.

Plasmid analysis has been used for epidemiologic studies of several outbreaks involving aminoglycosides-resistant and *β*-lactamase producing enterococci [43, 44]. Studies by Dutka-Malen et al. [45] of glycopeptide-resistant enterococci isolates in hospitals in Europe and the United States concluded that the spread of high level resistance (*van*A phenotype) is due to dissemination of a gene rather than a bacterial clone or a single plasmid. Many attempts have been made to show the ability of enterococci to transfer genes encoding for antibiotic resistance with the same or different enterococci species, as well as to other members of other bacteria genera [36]. The plasmid carriage by* E*.* faecium* identified in this present study appears to be low, limiting the usefulness of plasmid typing of these isolates.

The significantly higher prevalence of VRE in the two regional hospitals, “C” and “D” hospitals, suggests that these hospitals may be at a greater risk of VRE dissemination as there was evidence of high consumption and usage of antibiotics in these institutions [6].

## 5. Conclusion

This analysis indicates that the prevalence of VRE is low among clinical isolates in the country. Our findings confirm the potential for interhospital spread of VRE and highlight the importance of strengthening the practice of appropriate infection control protocols or early implementation in hospitals in Trinidad and Tobago.

The PFGE, RAPD, and Rep-PCR proved useful in typing vancomycin-resistant enterococci isolates from Trinidad and Tobago. A high prevalence of the* esp* gene was seen among the polyclonal VRE infection isolates and molecular analysis suggests that intra- and interhospital spread of vancomycin-resistant enterococci clone carrying* van*A elements seem to be the main mechanism of vancomycin-resistance dissemination in Trinidad and Tobago.

Continued surveillance activities for VRE are needed to detect early occurrence, dissemination, and corresponding increase in VRE prevalence locally. Further studies such as determination of sequence typing (ST) by multilocus sequence typing (MLST) or multiple-locus variable number tandem repeat analysis (MLVA) are warranted, and carriage rate of VRE among individuals in the country should be investigated.

## Figures and Tables

**Table 1:** Showing various pulsed-field gel electrophoresis (PFGE) groups of 45 vancomycin-resistant enterococcal isolates from regional hospitals in Trinidad and Tobago (%).

Source	*N*	*E. faecium *					*E. faecalis*
		PFGE-1	PFGE-2	PFGE-3	PFGE-4	PFGE-5	PFGE
UGT	17 (38)	4	1	6	2	4	7
SSTI	19 (42)	6	4	5	0	4	0
Blood	1	0	1	0	0	0	0
GIT^*∗*^	1	1	0	0	0	0	0
Total	38	11 (29)	6 (16)	11 (29)	2 (5)	8 (21)	7

*N* = number of isolates distribution; PFGE = pulsed-field gel electrophoresis pattern signifying the same clone; UGT = urogenital tract; SSTI = skin and soft tissue infections; GIT^*∗*^ = gastrointestinal tract, and this sole isolate was from the peritoneal fluid of a patient who had peritonitis. All seven vancomycin-resistant *E. faecalis* had an identical PFGE pattern indicating they belong to the same clone.
